# Lower limb lymphedema and cellulitis as a complication of COVID‐19 vaccine: A case report

**DOI:** 10.1002/ccr3.6317

**Published:** 2022-12-15

**Authors:** Ahmad Hosseinzadeh, Kamyar Ebrahimi, Reza Shahriarirad, Farzad Dalfardi

**Affiliations:** ^1^ Thoracic and Vascular Surgery Research Center Shiraz University of Medical Sciences Shiraz Iran; ^2^ School of Medicine Shiraz University of Medical Sciences Shiraz Iran

**Keywords:** cellulitis, COVID‐19, lymphedema, vaccination

## Abstract

A 68‐year‐old man without complications following his first dose of Sinopharm (BBIBP‐CorV) COVID‐19 vaccine developed left foot and ankle edema, extending to his left leg 3 days after his second dose. Color‐Doppler sonography and lymphoscintigraphy showed extensive soft tissue swelling and fat edema in both legs, proposing lymphatic drainage disorder.

## Graphical abstract

Physicians should consider secondary lymphedema among the complications of the COVID‐19 vaccine. Prolonged inflammation in lymphedema patients might compromise lymphatic function and increase edema.

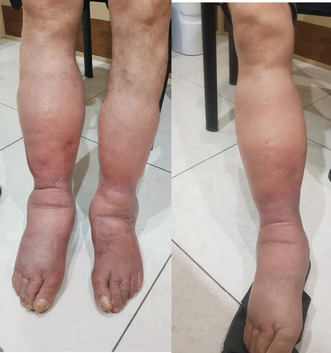

## 1 BACKGROUND

With the ongoing battle against the coronavirus disease 2019 (COVID‐19), there is a need for rapid vaccination in order to manage this disease.

Both the short‐term and the long‐term side effects of the available vaccines are yet to be revealed. Therefore, gathering and reporting evidence in this regard is vital to increase awareness of probable complications. Secondary lymphedema is a condition that develops as a result of a disease, trauma, or an iatrogenic process that damages the lymphatic system, such as surgery or radiation.^1^, ^2^ Secondary lymphedema can cause edema in the clinic.^3^ Vaccinations have been linked to lymphoedema, but there is no reliable scientific evidence to back up or refute this claim.^4^ Here, we report a case of secondary lymphedema following the second dose of Sinopharm (BBIBP‐CorV) COVID‐19 vaccination.

## 2 CASE PRESENTATION

The patient is 68‐year‐old man with a history of COVID‐19 infection in July 2021. About 75% of pulmonary parenchyma was involved. As a result, the patient was hospitalized and treated with a course of Remdesivir. About 2 months after his COVID‐19 infection, the patient received the first dose of the Sinopharm (BBIBP‐CorV) COVID‐19 vaccine. There were no complications or any unusual symptoms after the first injection.

A month after the first dose, the patient received the second dose of the Sinopharm COVID‐19 vaccine. Three days after the second dose, the patient developed edema in his left foot and ankle. This edema then extended to his entire left leg.

Due to these events, the patient visited a hospital where he underwent Color‐Doppler Sonography (CDS), which showed extensive soft tissue swelling and fat edema in both legs, proposing a lymphatic drainage disorder. There were no signs of clot or deep vein thrombosis in the legs, and blood flow was normal in the popliteal and femoral veins, with good compressibility. A small baker's cyst was also present in the right popliteal cavity.

**FIGURE 1:**
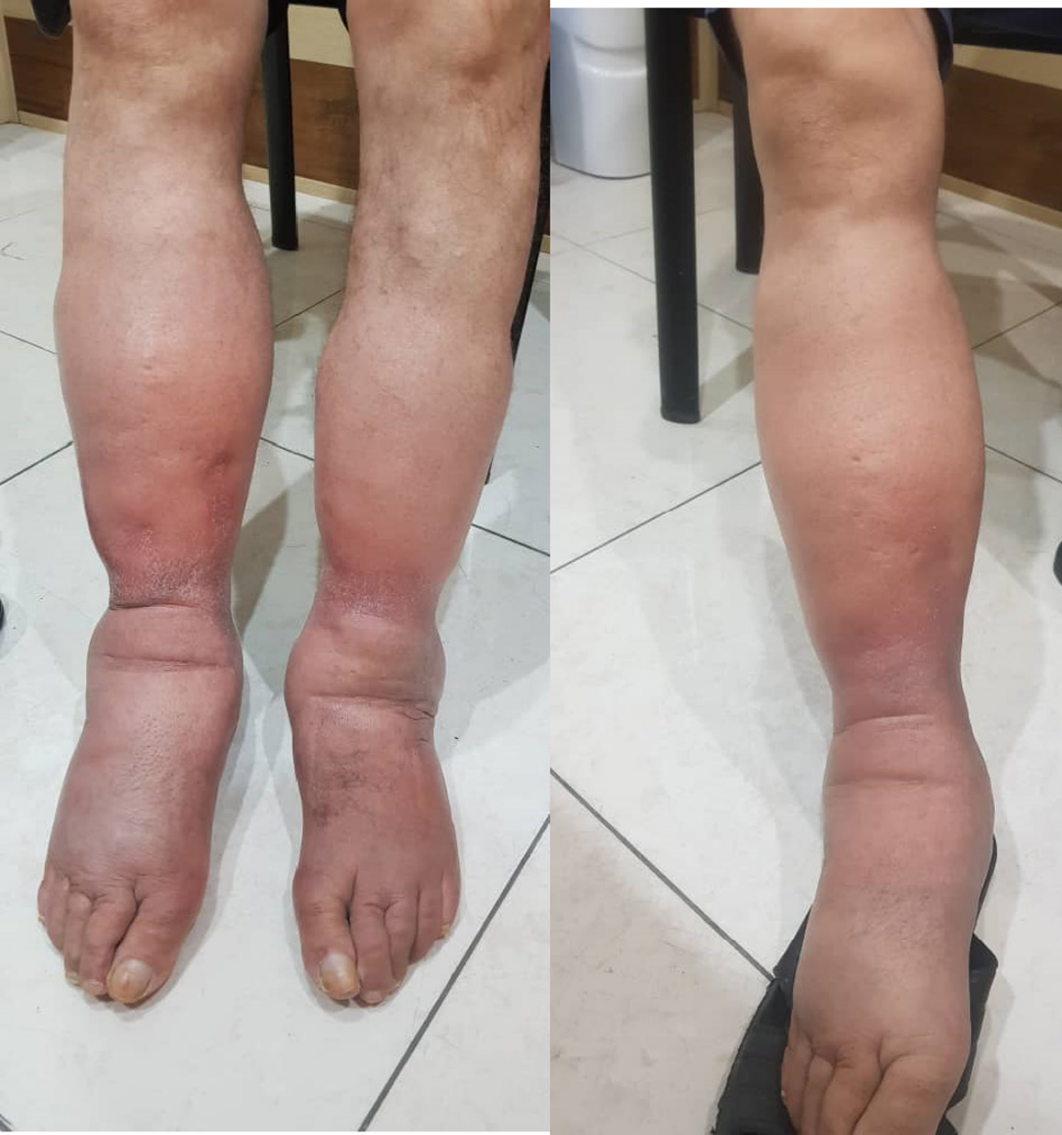
Bilateral pitting edema; limited below the knees

Five days after the initiation of edema in his left leg, the patient developed edema in his right foot and ankle. The edemas in both feet had redness and were warm to the touch. Due to the unexplainable edema and redness of both legs, the patient was referred to a vascular surgeon. Upon visiting, the patient had bilateral lower extremity edema. The edema was pitting and did not extend to the knees or higher. There was no ischemia, ecchymosis, arthritis, or any sign of articular trauma. All the distal lower extremity pulses were detected and were normal (Figure 1).

The patient stated he had no history of diabetes, heart diseases, hypertension, and thyroid diseases. There was also no history of rheumatologic diseases or any previous deep vein thrombosis. The patient also stated that he had a right knee injury 4 years ago, which was completely healed and did not cause any problems for him since. There is also a history of inguinal hernia from 35 years prior. The hernia was successfully repaired. The relation between the past inguinal hernia and the findings of this study is yet to be discovered. The patient has been an active individual who hikes daily and climbs mountains weekly.

Lymphangitis and cellulitis were the most probable differential diagnosis. Therefore, the patient was given an oral antibiotic (Levofloxacin 500 mg daily) for 2 weeks.

Laboratory data demonstrated high levels of inflammatory factors, including Erythrocyte sedimentation rate of 68 mm/h and positive C‐Reactive Protein. Albumin levels were normal and 3.9 g/dl. The patients' other tests were normal, including complete blood count, liver enzyme levels, kidney function, thyroid function, and rheumatoid factors. Echocardiography was also unremarkable.

The repeated CDS showed no sign of deep vein thrombosis, cloth, or varicose veins. The small baker cyst mentioned in the earlier CDS was also present. All veins were compressible. There were also no signs of obstruction or narrowing of the arteries. No insufficiencies in the Saphenofemoral junction or Saphenopopliteal junction were seen. The CDS also showed subcutaneous edema in both the ankle and foot of both lower extremities.

These results confirmed the lymphangitis and cellulitis diagnosis. The patient received Levofloxacin (500 mg daily), Aspirin (80 mg daily), and Apixaban (2.5 mg two times per day) for 2 weeks.

Ensuing, the redness and the warmth were mostly resolved, but the edema of the legs was unchanged. The patient received a Lymphoscintigraphy with two injections of 0.5mCi99m‐Tc‐phytate in the first interdigital web of both feet. This study revealed lymph drainage insufficiency in the right inguinal area and the lower part of the trunk. There were no findings indicating any lymph drainage insufficiency in the left groin.

Our patient also received a computed tomography (CT) scan of the abdomen, pelvis, and leg. The CT scan showed no sign of tumor or lymphadenopathy in the abdominal or para‐aortic areas.

Considering the clinical features of this edema (foot hump and edema in feet, ankles, and legs), we can conclude that the patient was suffering from the early stages of lymphedema. Bearing in mind that the patient's history of vaccination was just 3 days before the symptoms appeared, and there are papers on similar symptoms after COVID‐19 infections, it can be concluded that this lymphedema is a probable symptom of COVID‐19 vaccination.

## 3 DISCUSSION

COVID‐19 patients can develop a variety of clinical symptoms. The most prominent is an increased risk of venous thromboembolism, which includes deep venous thrombosis (DVT).^5^ Recent findings imply that vaccination, and the illness, can cause cerebral venous thrombosis, portal venous thrombosis, and DVT, all of which are major issues.^5^, ^6^

In several studies, Sinopharm has been shown to be safe and well‐tolerated, with all vaccinated patients reporting a strong humoral immune response.^7^, ^8^ The distribution of these vaccinations to lymph nodes via dendritic cells determines their efficiency. Some antigens can be directly transmitted to lymph nodes.^9^ Some individuals, particularly those with afferent lymphatics or lymph nodes, are vulnerable to vaccine‐induced lymphadenopathy during this phase.^10^ As a result, the Centers for Disease Control and Prevention (CDC) recommends that individuals at risk of lymphedema get COVID‐19 immunization on the opposite arm or leg. Our case demonstrates the first report of lymphadenitis following Sinopharm vaccination in a male individual without any significant past medical history. This complication has also been previously reported following the BNT16b2 Pfizer vaccine.^11^

Cellulitis following COVID‐19 has also been rarely reported in the literature.^12^, ^13^ A study from Japan also reported four patients with secondary lymphedema on lower limbs developed cellulitis following the COVID‐19 mRNA vaccination.^14^ According to a study regarding cutaneous allergic responses following vaccination, 63% of individuals had symptoms after the second vaccination dose but not after the first, similar to our case^15^; however, some reports state cutaneous responses following the initial vaccine.^14^ In our case, the increase in inflammatory markers along treatment with oral antibiotics was beneficial in confirming the diagnosis of cellulitis rather than an allergic response.

COVID‐19 infection and detection among health care professionals is also an important issue, which has been relatively controlled with mass vaccination programs.^16^, ^17^, ^18^, ^19^, ^20^, ^21^, ^22^, ^23^ However, BNT162b2 mRNA vaccination in medical professionals resulted in cellulitis in 0 out of 1245 subjects.^24^ Out of 1116 subjects who received the mRNA‐1273 vaccination, no one experienced cellulitis.^25^ According to some reports,^26^ cellulitis only developed at the site of the local vaccination. In a report by Okazaki et al.,^14^ only two of the four patients had CRP data; in both cases, the levels were elevated, and the eosinophil counts were within normal limits. According to a study on cutaneous allergic reactions following vaccinations, 63% of cases experienced reactions only after the second vaccination and not for the first.^15^ In contrast, after receiving the first vaccination in Okazaki et al. study,^14^ three out of four patients experienced skin reactions. Since those who received the first vaccination dose were sensitized and then experienced an immune response after receiving the second dose, they concluded that the reaction was not allergic. Additionally, the success of oral antibiotic therapy alone supported the diagnosis of cellulitis rather than an allergic reaction.

There is a proposed mechanism for frequent activation of cellulitis at lymphedema lesions as a side effect of COVID‐19 mRNA vaccines. The way this mechanism may work is by hyaluronan accumulation. Hyaluronan can build up in lymphedema lesions, causing inflammation to deteriorate. This substance is commonly used as a dermal filler. Therefore, COVID‐19 mRNA vaccination has been linked to inflammatory responses to hyaluronan and dermal fillers.^15^, ^27^ Furthermore, lymphatics are the primary pathway for hyaluronan drainage.^28^, ^29^ Hyaluronan can accumulate in tissues with dysfunctional lymphatics, such as lymphedema and tumors.^28^, ^29^ Overall, following COVID‐19 mRNA immunization, hyaluronan‐accumulating tissues may become highly immunogenic. These tissues develop cellulitis. This cellulitis is a response to the invasion of pathogens. Since these pathogens are low‐immunogenic, hosts usually do not respond to these pathogens. Inflammation changes lymphatic shape, impairs drainage performance, and worsens lymphedema.^30^ In cancer models, lymphatics with atypical shapes also reduced immune function.^29^ As a result, they might compromise immune function in infectious disorders like cellulitis. As a result, immediate treatment of cellulitis at lymphedema lesions is vital.

## 4 CONCLUSION

Secondary lymphedema should be considered among the complications of the COVID‐19 vaccine. This case report demonstrates that individuals who have received a COVID‐19 vaccine may get swelling of the lower extremities for no apparent reason. In these cases, the possibility of DVT should be ruled out first. However, we cannot ignore the possibility of transitory lymphedema related to lymphatic system failure without atypical ultrasonographic results. Prolonged inflammation in lymphedema patients might compromise lymphatic function and increase edema. As a result, we should consider the prevention and rapid management of cellulitis and utilize intensive skincare and antibiotic therapy at the time of vaccination. Because most of the symptoms are modest, short‐term rehabilitation treatment is beneficial alone. Both patients and physicians must be aware of possible complications in the event of widespread immunization since new, unexpected, or significant side effects must be reported if they are suspected.

## AUTHOR CONTRIBUTIONS

A.H. and F.D. carried out the diagnosis. K.E. and R.S. collected the data and drafted the manuscript. All authors read and approved the final version of the manuscript.

## CONFLICT OF INTEREST

The authors declare that they have no competing interests.

## ETHICAL APPROVAL

The present study was approved by the Medical Ethics Committee of Shiraz University of Medical Sciences.

## CONSENT

The purpose of this report was completely explained to the participant and was assured that his information will be kept confidential by the researchers. A written consent form was also obtained from the participant for the publication of this report and any accompanying images. A copy of the written consent is available for review by the Editor of this journal.

## Data Availability

All data regarding this case report has been reported in the manuscript. Please contact the corresponding author in case of requiring any further information.

## References

[1] Warren AG , Brorson H , Borud LJ , Slavin SA . Lymphedema: a comprehensive review. Ann Plast Surg. 2007;59(4):464‐472. 17901744 10.1097/01.sap.0000257149.42922.7e

[2] Tretbar LL , Morgan CL , Byung‐Boong L , Simonian SJ , Blondeau B . Lymphedema. Springer; 2008.

[3] Kerchner K , Fleischer A , Yosipovitch G . Lower extremity lymphedema update: pathophysiology, diagnosis, and treatment guidelines. J Am Acad Dermatol. 2008;59(2):324‐331. 18513827 10.1016/j.jaad.2008.04.013

[4] Lee TS , Baumgart KW . Vaccines and risk of lymphoedema–a case report of a breast cancer patient. Aust Fam Physician. 2012;41(6):404‐406. 22675681

[5] Sharifian‐Dorche M , Bahmanyar M , Sharifian‐Dorche A , Mohammadi P , Nomovi M , Mowla A . Vaccine‐induced immune thrombotic thrombocytopenia and cerebral venous sinus thrombosis post COVID‐19 vaccination; a systematic review. J Neurol Sci. 2021;428:117607. 34365148 10.1016/j.jns.2021.117607 PMC8330139

[6] Haakonsen HB , Nystedt A . Deep vein thrombosis more than two weeks after vaccination against COVID‐19. Tidsskr Nor Legeforen. 2021:141. 10.4045/tidsskr.21.0274 33928773

[7] Wang H , Zhang Y , Huang B , et al. Development of an inactivated vaccine candidate, BBIBP‐CorV, with potent protection against SARS‐CoV‐2. Cell. 2020;182(3):713‐721 e9. 32778225 10.1016/j.cell.2020.06.008 PMC7275151

[8] Cohen J . Dosing debates, transparency issues roil vaccine rollouts. Science. 2021;371:109‐110. 33414197 10.1126/science.371.6525.109

[9] Mahase E . Covid‐19: schedule breast screening before vaccine or 4 to 6 weeks after to avoid false positives, says guidance. BMJ. 2021;372:n617. 33658188 10.1136/bmj.n617

[10] Ozutemiz C , Krystosek LA , Church AL , et al. Lymphadenopathy in COVID‐19 vaccine recipients: diagnostic dilemma in oncologic patients. Radiology. 2021;300(1):E296‐E300. 33625300 10.1148/radiol.2021210275 PMC7909072

[11] Chung JH , Sohn SM , Yoo HJ , Yoon ES , Park SH . Transient lower extremity lymphedema following COVID‐19 vaccination: a case report. Medicine (Baltimore). 2021;100(48):e28092. 35049235 10.1097/MD.0000000000028092 PMC9191610

[12] Klein NP , Lewis N , Goddard K , et al. Surveillance for adverse events after COVID‐19 mRNA vaccination. JAMA. 2021;326(14):1390‐1399. 34477808 10.1001/jama.2021.15072 PMC8511971

[13] Sun Q , Fathy R , McMahon DE , Freeman EE . COVID‐19 vaccines and the skin: the landscape of cutaneous vaccine reactions worldwide. Dermatol Clin. 2021;39(4):653‐673. 34556254 10.1016/j.det.2021.05.016 PMC8165093

[14] Okazaki T , Matashiro M , Kodama G , Tshubota T , Furusawa Y , Izumi S‐I . Frequent onsets of cellulitis in lower limbs with lymphedema following COVID‐19 mRNA vaccination. Vaccines (Basel). 2022;10(4):517. 35455266 10.3390/vaccines10040517 PMC9025572

[15] McMahon DE , Amerson E , Rosenbach M , et al. Cutaneous reactions reported after Moderna and Pfizer COVID‐19 vaccination: a registry‐based study of 414 cases. J Am Acad Dermatol. 2021;85(1):46‐55. 33838206 10.1016/j.jaad.2021.03.092 PMC8024548

[16] Parvar SY , Ghamari N , Pezeshkian F , Shahriarirad R . Prevalence of anxiety, depression, stress, and perceived stress and their relation with resilience during the COVID‐19 pandemic, a cross‐sectional study. Health Sci Rep. 2022;5(1):e460. 35024455 10.1002/hsr2.460 PMC8733840

[17] Sabetian G , Azimi A , Kazemi A , et al. Prediction of patients with COVID‐19 requiring intensive care: a cross‐sectional study based on machine‐learning approach from Iran. Indian J Crit Care Med. 2022;26(6):688‐695. 35836646 10.5005/jp-journals-10071-24226 PMC9237161

[18] Sabetian G , Moghadami M , Haghighi LHF , et al. COVID‐19 infection among healthcare workers: a cross‐sectional study in Southwest Iran. Virol J. 2021;18(1):1‐8. 33731169 10.1186/s12985-021-01532-0 PMC7968574

[19] Sabetian G , Shahriarirad S , Moghadami M , et al. High post‐infection protection after COVID‐19 among healthcare workers: a population‐level observational study regarding SARS‐CoV‐2 reinfection, reactivation, and re‐positivity and its severity. Res Sq. 2021. doi:10.21203/rs.3.rs-772662/v1 PMC11053253 38680224

[20] Shafiekhani M , Niknam T , Tara SA , et al. COVID‐19 versus applied infection control policies in a major transplant Center in Iran. Res Sq. 2021. doi:10.21203/rs.3.rs-757719/v1. PMC9969367 36849978

[21] Shahriarirad R , Erfani A , Ranjbar K , Bazrafshan A , Mirahmadizadeh A . The mental health impact of COVID‐19 outbreak: a Nationwide Survey in Iran. Int J Ment Health Syst. 2021;15(1):19. 33640006 10.1186/s13033-021-00445-3 PMC7913044

[22] Shahriarirad R , Fallahi MJ . TB and the COVID‐19 pandemic: brothers in arms against lung health. Int J Tuberc Lung Dis. 2020;24(10):1126‐1127. 33126954 10.5588/ijtld.20.0449

[23] Shahriarirad R , Sarkari B . COVID‐19: clinical or laboratory diagnosis? A matter of debate. Trop Doct. 2021;51(1):131‐132. 32762302 10.1177/0049475520945446

[24] Kadali RAK , Janagama R , Peruru S , Malayala SV . Side effects of BNT162b2 mRNA COVID‐19 vaccine: a randomized, cross‐sectional study with detailed self‐reported symptoms from healthcare workers. Int J Infect Dis. 2021;106:376‐381. 33866000 10.1016/j.ijid.2021.04.047 PMC8049195

[25] Kadali RAK , Janagama R , Peruru S , et al. Non‐life‐threatening adverse effects with COVID‐19 mRNA‐1273 vaccine: a randomized, cross‐sectional study on healthcare workers with detailed self‐reported symptoms. J Med Virol. 2021;93(7):4420‐4429. 33822361 10.1002/jmv.26996 PMC8250701

[26] Ramalingam S , Arora H , Lewis S , et al. COVID‐19 vaccine‐induced cellulitis and myositis. Cleve Clin J Med. 2021;88(12):648‐650. 34857596 10.3949/ccjm.88a.21038

[27] Munavalli GG , Guthridge R , Knutsen‐Larson S , Brodsky A , Matthew E , Landau M . COVID‐19/SARS‐CoV‐2 virus spike protein‐related delayed inflammatory reaction to hyaluronic acid dermal fillers: a challenging clinical conundrum in diagnosis and treatment. Arch Dermatol Res. 2022;314(1):1‐15. 33559733 10.1007/s00403-021-02190-6 PMC7871141

[28] Roberts MA , Mendez U , Gilbert RJ , Keim AP , Goldman J . Increased hyaluronan expression at distinct time points in acute lymphedema. Lymphat Res Biol. 2012;10(3):122‐128. 22984909 10.1089/lrb.2012.0001 PMC3444763

[29] Tsukita Y , Okazaki T , Ebihara S , et al. Beneficial effects of sunitinib on tumor microenvironment and immunotherapy targeting death receptor5. Onco Targets Ther. 2019;8(2):e1543526. 10.1080/2162402X.2018.1543526 PMC6343781 30713805

[30] Nihei M , Okazaki T , Ebihara S , et al. Chronic inflammation, lymphangiogenesis, and effect of an anti‐VEGFR therapy in a mouse model and in human patients with aspiration pneumonia. J Pathol. 2015;235(4):632‐645. 25348279 10.1002/path.4473

